# Uric acid lowering improves insulin sensitivity and lowers blood pressure: a meta-analysis of randomized parallel-controlled clinical trials

**DOI:** 10.4314/ahs.v21i1.13

**Published:** 2021-03

**Authors:** Qunchuan Zong, Guanyi Ma, Tao Wang

**Affiliations:** Division of Orthopedic Surgery, the Affiliated Hospital of Qinghai University, 810001, Xining, Qinghai Province, China

**Keywords:** Hyperuricemia, uric acid lowering treatment, β-cell function, insulin sensitivity

## Abstract

**Objectives:**

This meta-analysis aimed to investigate whether uric acid lowering treatment can improve β-cell function and insulin sensitivity.

**Methods:**

PubMed, Cochrane Library, EMBASE and China Biology Medicine were searched up to March 1, 2020. Randomized controlled clinical trials of urate lowering therapy in hyperuricemia patients were included in meta-analysis. Effect size was estimated as mean difference with 95% confidence interval (CI).

**Results:**

Our search yielded 7 eligible trials with 503 participants. This meta-analysis showed that uric acid-lowering therapy decreased fasting insulin -1.43 µIU/ml (weighted mean differences (WMD, 95% CI -2.78 to -0.09), homeostasis model assessment of insulin resistance -0.65 (WMD, 95% CI -1.05 to -0.24), systolic blood pressure -2.45 mm Hg (WMD, 95%CI -4.57 to -0.33) and diastolic blood pressure -3.41 mm Hg (WMD, 95%CI -3.87 to -2.95). However, the treatment had no significant effect on fasting plasma glucose (WMD -0.19 mmol/L, 95%CI -0.42 to 0.05), homeostasis model assessment of β-cell function index (WMD -0.02, 95%CI -0.28 to 0.24), total cholesterol (WMD 0.18 mg/dl; 95%CI, -1.39 to 1.75) and triglyceride (WMD 3.15 mg/dl, 95% CI -9.83 to 16.14).

**Conclusion:**

Uric acid-lowering therapies might improve insulin sensitivity and lower blood pressure, but had no significant effect on HOMA-β and serum lipids.

## Background

Metabolic syndrome is a cluster physiological and anthropometric abnormalities of nutrients metabolism, such as hyperglycemia, hyperuricemia and hyperlipidemia^1^,^2^. The metabolism of three major nutrients are closely linked. Hyperuricemia could contribute to abnormal glucose metabolism, insulin resistance (IR), even pancreatic β-cell death^3^–^7^. Substantial data from epidemiologic and experimental studies indicate an emerging association between hypruricemia, type 2 diabetes mellitus (T2DM), and cardiovascular-related diseases.^8^ Several studies revealed that hyperuricemia may be an independent risk factor for the development of T2DM 9–13, which suggest a substantial implication for a correlation between uric acid concentration and insulin resistance (or insulin sensitivity) 13–15. Also, hyperuricemia is substantially implicated in cardiovascular risks,^16^–^18^ vascular complications,^19^ the further long-term cardiovascular events^13^ and mortality^19^ in T2DM patients. Pharmacologic agents lowering serum UA proved to play a promising role in the management of T2DM and CVD related disease 8. Asymptomatic hyperuricemia is a common entity faced by physicians in day to day practice. Although there are clear recommendations on the treatment of gout with urate lowering therapy (ULT), the management of asymptomatic hyperuricemia remains controversial, especially in diabetic patients with asymptomatic hyperuricemia. Based on above observation, we conduct a meta-analysis to investigate whether- LT might be helpful for diabetes patients.

## Materials and methods

### Design

The meta-analysis followed the Preferred Reporting Items for Systematic Reviews and Meta-Analyses (PRISMA) guidelines for meta-analyses of interventional studies.

### Data sources and search strategy

English and Chinese language publications were identified from MEDLINE, EMBASE, Cochrane Library and Chinese Biomedical Literature Database (CBM) from inception to May 2016. Search strategy was as follows: (“Hyperuricemia/drug therapy” [Mesh] AND “Diabetes Mellitus” [Mesh]) OR ((diabetic OR glucose) AND (uric acid)) AND (randomized controlled trial [pt] OR controlled clinical trial [pt] OR randomized [tiab] OR placebo [tiab] OR clinical trials as topic [mesh: noexp] OR randomly [tiab] OR trial [ti]) NOT (animals [mh] NOT humans [mh]).

### Study selection

Eligible studies fell into four categories, including (1) randomized controlled trials (RCTs); (2) participates were hyperuricemia patients in adults with or without diabetes, and hyperuricemia is defined with the World Health Organization (WHO) diagnostic criteria; (3) intervene with hypouricemic agents except losartan and fenofibrate (because of their effect on blood pressure and insulin sensitivity); (4) reported at least fasting plasma glucose (FPG), fasting insulin (FINS) and homeostasis model assessment of insulin resistance (HOMA-IR) outcome data.

Meanwhile, we excluded the studies that were cross-sectional studies, cohort studies or studies on animals; or that were duplicated. If the same population was reported in more than one studies, we included the one with the most complete data.

### Data extraction and synthesis

For each eligible trial, we extracted data on name of the first author, year of publication, study design, participants' characteristics, sample size for each group, intervention period, contents of intervention and control conditions, as well as main study outcomes.

Outcome measures were classified into three aspects: (1) β-cell function as reflected changes in FPG, FINS and homeostasis model assessment of β-cell function (HOMA-β); (2) the level of IR was reflected as change in HOMA-IR; (3) cardiovascular risk factors including changes in total cholesterol (TC), triglyceride (TG) and blood pressure (BP).

### Quality assessment

The quality of the individual studies was assessed by the Cochrane Risk of Bias tool^20^. The categories were random sequence generation, allocation concealment, blinding (participants, study personnel and outcome assessment), incomplete outcome data addressed, selective outcome reporting and other sources of bias. Quality was rated as ‘high’ if at least the first three criteria (adequate sequence generation, allocation concealment and blinding) were fulfilled and not more than one of the others was rated ‘unclear’. Quality was rated as ‘low’ if these first three or any other four criteria were rated as unclear or inadequate. All the otherwere rated as ‘medium’ quality. Two investigators independently evaluated the above risk of bias domains and consensus was achieved through a third reviewer.

The pooled analyses were performed using STATA version 12.0. The Cochrane Q and I^2^ statistics was carried out to assess heterogeneity across studies. For the Q statistic, a level of P value ≤ 0.10 was considered statistically significant for heterogeneity; for I^2^, values of 25, 50 and 75% indicate low, moderate and high levels of heterogeneity, respectively^21^, ^22^. Weighted mean difference (WMD) and 95% CIs between ULT group and the control were calculated as the effect size for continuous outcomes^23^. We performed primary analyses using a random effects model that adequately accounts for between-study variability. Sensitivity analyses and subgroup analyses were also performed when required. Furthermore, potential publication bias was assessed by Begg's 24 funnel plot and Egger's^25^ regression test and a two-sided P-value < 0.05 was considered statistically significant.

## Results

### Search results

**Figure 1:**
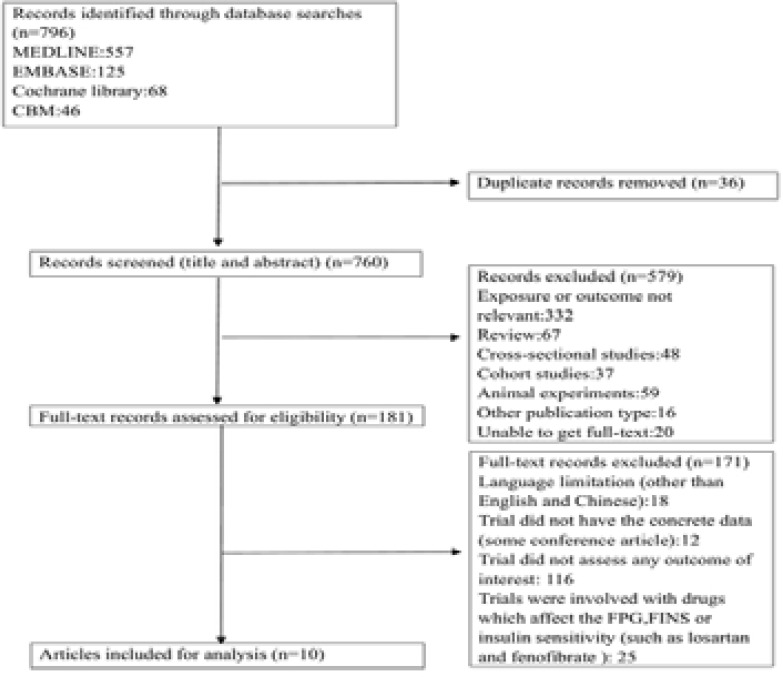
Flowchart of the study selection.

A flowchart of the study selection process was presented in Figure 1. We conducted a literature search in PubMed, EMBASE and the Cochrane library from inception to October 25, 2019. We initially searched a total of 796 citations from the PubMed database, EMBASE, Cochrane Library and CBM, of which 615 citations were excluded after the first screening based on titles and abstracts. They were excluded for the following main reasons: a total of 36 studies were duplicated and exposure or outcomes of 332 studies were not relevant. There were 227 studies reported as cross-sectional study, cohort study or other publication type. And 20 were conference article or other types which were short of full and exact data. One hundred and eighty-one full-text articles were reviewed for detailed assessment. Eighteen articles of them were published neither in English or Chinese and 116 trials didn't assess any outcomes relevant to β-cell function or insulin sensitivity. Twelve articles didn't have the detailed information (change value or 95%CI of primary outcome). In 25 trials, agents, such as losartan and fenofibrate, had effect on both SUA and β-cell function or insulin sensitivity. Finally, 7 eligible citations were included in our meta-analysis^26^–^32^. Then, manual searching was conducted according to the references of relevant acquired articles. The search was later updated to March 1, 2020. No newly identified study was included in the analyses.

### Study characteristics

The characteristics of the included studies were presented in Table 1. Seven randomized controlled-trials included a total of 503 participants (mean age 50±10.50 years) 26–32. The duration of follow-up period ranged from 2 weeks to 156 weeks, with a median of 60 weeks. Of these, six studies were intervened with allopurinol^26^–^31^ and one with benzbromarone^32^. All the individual studies were based on general population of predominantly middle-aged or older participants with hyperuricemia, except one based on non-hyperuricemia but induced to hyperuricemia by excessive fructose intake^27^. Then the hyperuricemia patients induced by excessive fructose intake were randomized to two groups: one was given allopurinol when the SUA was greater than 420 µmol/L, the other received no treatment. Three of these studies were carried out in the patients of hyperuricemia with either T2DM or impaired glucose tolerance (IGT) 26, 29, 31.

**Table 1:** Descriptive characteristics of included articles

References	Participants	n	Age	Duration, design	Intervention	Control	Available Outcomes	Limitation
Szwejkowski et al [22], 2013	Patients with hyperuricemia and T2DM	66 (59 completers)	64.63±8.79 years	9 months, RCT	Allopurinol 600 mg twice per day (n=33)	Placebo (n=33)	Difference 600 mg allopurinol versus control (95% CI): UA (µmo/l): -251.00 [-313.02, -188.98] FPG (mmol/l): -0.43 [-2.39, 1.53] FINS (µU/ml): 4.77 [-4.27, 13.81]	Not adjusted for diabetes duration, kinds or dosage of hypoglycemic agents. Not aims to evaluate the effect of uric acid-lowering therapy on β-cell function or insulin sensitivity.
Perez-Pozo et al [23], 2010	Participants were administered fructose 200 g daily	83 (74 completers)	40.65 years (mean 51± 1.3 years)	2 weeks, RCT	Allopurinol 200 mg/d (n=38)	No treatment (n=36)	Difference 100mg allopurinol versus control (95% CI) UA (µmo/l): -178.00 [-182.29, -173.71] FPG (mmol/l) : 0.10 [0.07, 0.13] (µU/ml): -0.58 [-0.85,-0.31] HOMA-IR : -0.39 [-0.85, 0.07]	Participates were induced to hypeluricemia by fructose. Not adjusted for diabetes duration, kinds or dosage of hypoglycemic agents. Not to evaluate the effect of uric acid-lowering therapy on β-cell function or insulin sensitivity.
Takir et al [24], 2014	Patients with hyperuricemia	73	50.76 ± 13.78 years	3 months, RCT	Allopurinol 300 mg/d (n=40)	No treatment (n=33)	Difference 300 mg allopurinol versus control (95% CI): UA (µmo/l): -72.00 [-95.86, -48.14] FPG (mmol/l) :-0.40 [-0.61,-0.19] FINS (µU/ml): -2.00 [-3.99,-0.01] HOMA-IR : -3.40 [-5.22, -1.58]	Choice of allopurinol versus control was performed by the treating physician. Hence, it was not a pure randomization.
Liu et al [25], 2015	Patients with T2DM and hyperuricemia	176 (152 completers)	50.5 ± 10.49 years	3 years, RCT	Allopurinol (starting from 100 mg/day) adjust to SUA (n=88)	No treatment when the SUA was less than 476 µmol/L (n=88)	Difference allopurinol versus control (95% CI) UA (µmo/l): -138.00 [-142.13, -133.87] FPG (mmol/l) : 0.01 [-0.11, 0.13] FINS (µU/ml): -0.51 [-0.73, -0.29] HOMA-IR : -0.67 [-0.99, -0.34]	Open-label design and the lack of a placebo control. Not adjusted for diabetes duration, kinds or dosage of hypoglycemic agents. Not aims to evaluate the effect of uric acid-lowering therapy on β-cell function or insulin sensitivity.
Ding et al [26], 2012	Patients with hyperuricemia	60	48.0±11.7 years	3 ∼ 8 months (mean 4.3 months), RCT	Allopurinol 100 mg twice a day (n=30)	No treatment (n=30)	Difference 200mg allopurinol versus control (95% CI): UA (µmo/l): -140.6 [-2.63,-1.69] FPG (mmol/l) : 0.17 [-0.17,0.51] FINS (µU/ml): 7.28 [5.62, 8.94] HOMA-IR: 0.16 [-0.34, 0.67]	Unclear for random sequence generation or double-blind design. Not adjusted for kinds or dosage of hypoglycemic agents.
Le et al [27], 2013	Patients with hyperuricemia and IGT	40	44.65±2.27 years	6 months, RCT	allopurinol 100 mg three times a day (n=20)	Low purine and diabetes diet (n=20)	Difference 300mg allopurinol versus control (95% CI) UA (µmo/l): -203.00 [-221.97, -184.03] FPG (mmol/l) : -0.66 [-0.86, -0.46] FINS (µU/ml): -10.56 [-13.39, -7.73] HOMA-IR : -3.56 4.59, -2.53]	Unclear for random sequence generation or double-blind design. Not adjusted for kinds or dosage of hypoglycemic agents.
Ogino al [28], 2016	Patients with hyperuricemia	14	60±5 years	8 weeks, randomized crossover study	Benzbromarone 50 mg/d (n=7)	Placebo (n=7)	Difference 50 mg benzbromarone versus control (95% CI): UA (µmo/l): -128.90 [-156.88, -100.92] FPG (mmol/l): -0.33 [-0.56, -0.10] FINS (µU/ml): -7.80 [-9.26, -6.34] HOMA-IR : -1.22 [-1.72, -0.72]	Participates are patients with CHF and under the treatment of ACEIs, which may affect insulin sensitivity. Excluded diabetes mellitus and antidiabetic therapy. Short duration and small sample size. Not aims to evaluate the effect of uric acid-lowering therapy on β-cell function or insulin sensitivity.

Abbreviations: T2DM, Type 2 diabetes mellitus; RCT, randomized controlled trial; UA, uric acid; FPG. fasting plasma glucose; FINS, fasting insulin; HOMA-IR, homeostasis model assessment of insulin resistance; IGT, impaired glucose tolerance; CHF, chronic heart failure; ACEIs, angiotensin converting enzyme inhibitors.

### Quality of studies

The quality of the individual studies was assessed by the Cochrane Risk of Bias tool. Details of risk of bias assessment are shown in Table S1. The reporting quality was rated as ‘high’ in three of the studies^26^, ^29^, ^32^, ‘medium’ in one study^27^, and ‘low’ in three studies^28^, ^30^, ^31^.

**Table S1:** Quality assessment of included studies

Study	Random sequence generation	Allocation concealment	Blinding			Incomplete outcome data addressed	Free of selective reporting	Other sources of bias	Quantity

			Participants	Personnel	Outcome assessors				
Szwejkowskiet al [22], 2013	Yes	Yes	Yes	Yes	Yes	Yes	Yes	Not reported	High
Perez-Pozo et al [23], 2010	Yes	Yes	Yes	No	Not reported	Yes	Yes	Not reported	Medium
Takir et al [24], 2014	Unclear	Unclear	Unclear	Unclear	Not reported	Yes	Yes	Not reported	Low
Liu et al [25], 2015	Yes	Yes	Yes	Yes	Yes	Yes	Yes	Not reported	High
Ding et al [26], 2012	Unclear	Unclear	Unclear	Unclear	Not reported	Unclear	Yes	Not reported	Low
Le et al [27], 2013	Unclear	Unclear	Unclear	Unclear	Not reported	Yes	Yes	Not reported	Low
Ogino et al [28], 2016	Yes	Yes	Yes	Yes	Yes	Yes	Yes	Not reported	High

### Outcomes

**Figure 2:**
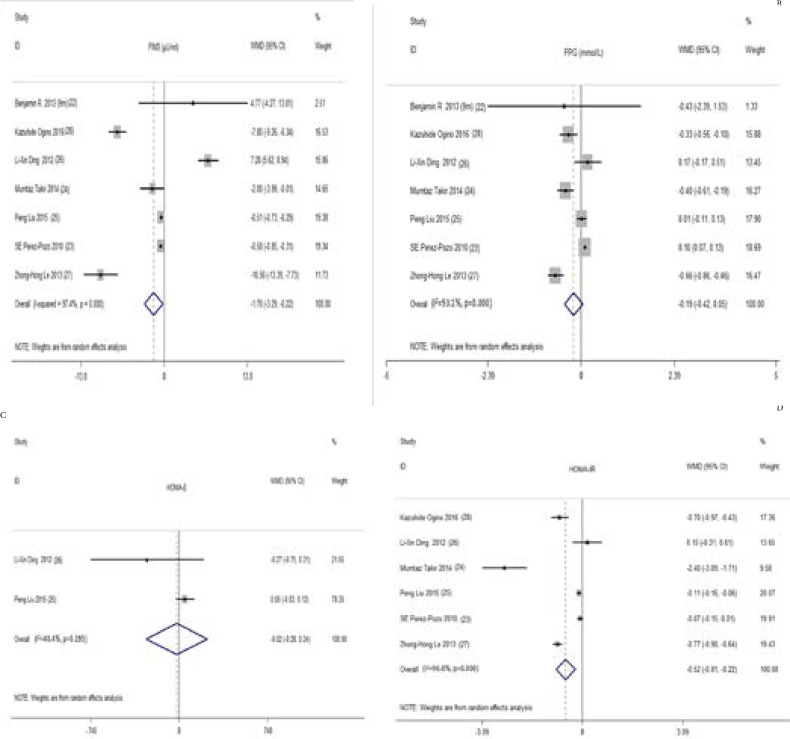
Forest plot and weighted mean differences (WMD) for effect of the uric acid lowering therapy on FINS (A), FPG (B), HOMA-β (C) and HOMA-IR (D). Abbreviations: Szwejkowski-1 is the result of 6 months' follow up. Szwejkowski-2 is the result of 9 months' follow up. WMD, weighted mean differences; CI, confidence interval; FINS, fasting insulin; HOMA-β, homeostasis model assessment of β-cell function index; HOMA-IR, homeostasis model assessment of insulin resistance.

FPG, FINS and HOMA-β (homeostasis model assessment of β-cell function). Seven studies26-32 with 503 participants in our main analyses for the changes in FPG and FINS from baseline pooled estimates showed hypouricemic treatment significantly decreased SUA (WMD, -165.52 µmol/L, 95% CI -189.91 to -141.14) by using a random effects model. At the same time, FINS was reduced by -1.43 µIU/ml (WMD, 95% CI -2.78 to -0.09) (Figure 2A). There were significant heterogeneities for the analyses of SUA (I^2^ = 98%; P = 0.00) and FINS (I^2^ = 97.4%; P = 0.00). However, hypouricemic therapy had no significant effect on FPG (WMD -0.19 mmol/L, 95% CI -0.42 to 0.05) (Figure 2B). Two studies with 236 participates measured HOMA-β and reported data that could be pooled in the analysis^29^, ^30^. Compared with the controls, there was no statistically significant change in HOMA-β (WMD - 0.02, 95% CI -0.28 to 0.24) (Figure 2C). Moderate heterogeneity was present between the two studies (I^2^ = 40.4%; P = 0.20). Insulin sensitivity (HOMA-IR) Six studies with 437 participants reported the change in HOMA-IR from baseline^27^–^32^. The treatment significantly mitigated HOMA-IR (WMD -0.65, 95%CI -1.05 to -0.24) (Figure 2D). Heterogeneity of the effect measures on HOMA-IR was detected (I^2^ = 96.6%; P =0.00).

**Figure 3:**
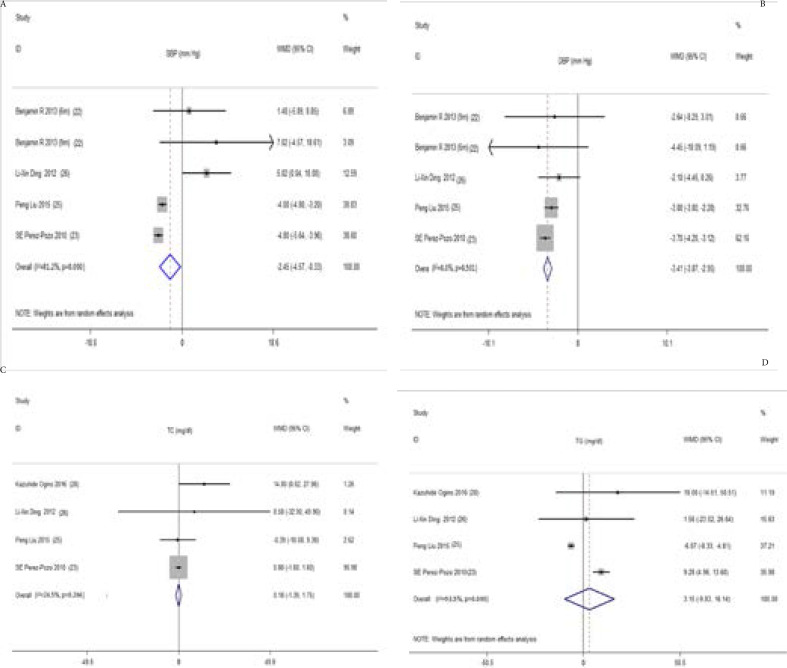
Forest plot and weighted mean differences (WMD) for effect of the uric acid lowering therapy on SBP (A), DBP (B), TC (C) and TG (D). Abbreviations: Szwejkowski-1 is the result of 6 months' follow up. Szwejkowski-2 is the result of 9 months' follow up; WMD, weighted mean differences; CI, confidence interval; SBP, systolic blood pressure; DBP, diastolic blood pressure; TC, total cholesterol; TG, triglyceride.

Cardiovascular risk factors (BP, TC and TG) Four studies involving 376 participants demonstrated that ULT was associated with a significant reduction of systolic blood pressures (SBP) (WMD -2.45 mm Hg, 95%CI -4.57 to -0.33) (Figure 3A) and diastolic blood pressures (DBP) (WMD -3.41 mm Hg, 95%CI -3.87 to -2.95) (Figure 3B) 26, 27, 29, 30. There was high level of heterogeneity for the analysis of SBP (I^2^ = 81.2%; P = 0.00), but no heterogeneity for DBP (I^2^= 0.00%; P = 0.50).

With regard to serum lipids, changes were pooled for 4 studies with 376 participants28, 29, 30, 32. It suggested that the hyperuricemia therapy had no significant reduction in TC (WMD 1.48 mg/dl, 95% CI -3.34 to 6.39) (Figure 3C) and TG (WMD 3.15 mg/dl, 95% CI -9.83 to 16.14) (Figure 3D) compared with the control. Low level of heterogeneity was observed in the analysis of TC (I^2^= 24.5%; P = 0.26). But the heterogeneity for the analysis of TG was severe (I^2^= 93.5%; P = 0.00).

### Subgroup and sensitivity analyses

Significant heterogeneities were observed in the primary outcome of FPG, FINS and HOMA-IR, therefore we performed further analyses. The subgroup analyses were stratified by (1) participates with or without hyperglycemia (normal glucose regulation, impaired glucose tolerance or diabetes mellitus), (2) dosage of hypouricemic agents (allopurinol ≥ 300 mg/d, allopurinol < 300 mg/d, benzbromarone 50 mg/d or adjust according to SUA), (3) mean UA at baseline (≥ 420 umol/L or < 420 umol/L), (4) reduction of SUA (≥ 150 umol/L or < 150 umol/L), (4) mean age of participants (mean age < 50 years or mean age < 50 years), (4) duration of the study (duration ≥ 6 months or duration < 6 months), (5) body mass index (BMI) (≥ 28 kg/m^2^ or < 28 kg/m^2^), (6) the number of participants (n > 60 or n 60) (Table S2–S4). Because of only 2 studies with data of HOMA-β, we didn't perform subgroup or sensitivity analysis.

**Table S2:** Subgroup analysis for FPG of various variables

Group	Number of studies	WMD (95% CI)	P for heterogeneity	I^2^ %
Total	7	-0.19 [-0.42, 0.05]	< 0.001	93.00
PG				94.00
NGR	4	-0.33 [-0.95, 0.29]	< 0.001	93.70
IGT or DM	3	-0.12 [-0.42, 0.19]	< 0.001	91.20
Dosage				
Allopurinol ≥ 300 mg/d	3	-0.53 [-0.74, -0.32]	0.217	34.60
Allopurinol < 300 mg/d	2	0.10 [0.07, 0.13]	0.688	0
Benzbromarone 50 mg/d	1	-0.33 [-0.56, -0.10]	-	-
Adjust according to SUA	1	0.01 [-0.11, 0.13]	-	-
Mean SUA at baseline				
≥ 420 umol/L	4	-0.17 [-0.61, 0.27]	< 0.001	92.00
< 420 umol/L	3	-0.20 [-0.58, 0.18]	< 0.001	94.00
Reduction of SUA				
≥ 150 umol/L	3	-0.29 [-0.99, 0.41]	< 0.001	96.00
< 150 umol/L	4	-0.13 [-0.38, 0.11]	< 0.001	85.00
Mean age				
≥ 50 years	5	-0.13 [-0.34, 0.07]	< 0.001	88.80
< 50 years	2	-0.26 [-1.07, 0.56]	< 0.001	94.10
BMI				
≥ 28	3	-0.15 [-0.62, 0.31]	< 0.001	91.00
< 28	3	-0.05 [-0.29, 0.20]	< 0.001	99.00
Duration				
≥ 6 months	3	-0.33 [-0.95, 0.29]	< 0.001	93.70
< 6 months	4.	-0.12 [-0.42, 0.19]	< 0.001	91.20
Number of participant				
>60	4	-0.07 [-0.28, 0.13]	< 0.001	87.10
≤60	3	-0.29 [-0.72, 0.14]	< 0.001	88.60

Abbreviations: PG, plasma glucose; NGR, normal glucose regulation; IGT, impaired glucose tolerance; DM, diabetes mellitus; SUA, serum uric acid; BMI, body mass index.

**Table S3:** Subgroup analysis for FINS of various variables

Group	Number of studies	WMD (95% CI)	P for heterogeneity	I^2^ %
Total	7	-1.43 [-2.78, -0.09]	< 0.001	97.00
PG				
NGR	4	-0.65 [-3.86, 2.56]	< 0.001	98.00
IGT or DM	3	-2.79 [-10.92, 5.34]	< 0.001	96.00
Dosage				
Allopurinol ≥ 300 mg/d	3	-3.52 [-10.68, 3.64]	< 0.001	94.00
Allopurinol < 300 mg/d	2	3.31 [-4.40, 11.01]	< 0.001	99.00
benzbromarone 50 mg/d	1	-7.80 [-10.31, -5.29]	-	-
Adjust according to SUA	1	-0.51 [-0.73, -0.29]	-	-
Mean SUA at baseline				
≥ 420 umol/L	4	0.35 [-13.48, 14.17]	< 0.001	90.00
< 420 umol/L	3	-1.27 [-2.02, -0.52]	< 0.001	98.00
Reduction of SUA				
≥ 150 umol/L	3	-2.82 [-10.90, 5.26]	< 0.001	96.00
< 150 umol/L	4	-0.49 [-3.53, 2.55]	< 0.001	97.00
Mean age				
≥50 years	5	-1.47 [-2.31, -0.63]	< 0.001	91.00
<50 years	2	-1.60 [-19.08, 15.88]	< 0.001	99.00
BMI				
≥ 28	3	-1.09 [-2.49, 0.30]	0.005	81.00
< 28	3	-0.20 [-6.43, 6.03]	< 0.001	98.00
Duration				
≥6 months	3	-2.79 [-10.92, 5.34]	< 0.001	96.00
<6 months	4.	-0.65 [-3.86, 2.56]	< 0.001	98.00
Number of participant				
>60	4	-0.77 [-1.23, -0.32]	< 0.001	73.00
≤60	3	-3.65 [-15.67, 8.36]	< 0.001	99.00

Abbreviations: PG, plasma glucose; NGR, normal glucose regulation; IGT, impaired glucose tolerance; DM, diabetes mellitus; SUA, serum uric acid; BMI, body mass index.

**Table S4:** Subgroup analysis for HOMA-IR of various variables

Group	Number of studies	WMD (95% CI)	P for heterogeneity	I^2^ %
Total	6	-0.65 [-1.05, -0.24]	< 0.001	98.00
PG				
NGR	4	-0.76 [-2.02, 0.51]	< 0.001	99.00
IGT or DM	2	-0.44 [-1.08, 0.21]	<0.001	99.00
Dosage				
Allopurinol ≥ 300 mg/d	2	-1.58 [-3.18, 0.02]	< 0.001	99.00
Allopurinol < 300 mg/d	2	-0.06 [-0.14, 0.02]	0.351	0
benzbromarone 50 mg/d	1	-0.70 [-1.30, -0.10]	-	-
Adjust according to SUA	1	-0.11 [-0.16, -0.06]	-	-
Mean SUA at baseline				
≥ 420 umol/L	3	-2.21 [-5.10, 0.69]	< 0.001	96.00
< 420 umol/L	3	-0.77 [-1.06, -0.47]	0.090	59.00
Reduction of SUA				
≥ 150 umol/L	2	-1.83 [-4.58, 0.91]	0.003	89.00
< 150 umol/L	4	-1.21 [-2.16, -0.27]	< 0.001	93.00
Mean age				
≥50 years	4	-0.79 [-1.29, -0.29]	< 0.001	99.00
<50 years	2	-0.34 [-1.24, 0.56]	< 0.001	93.10
BMI				
≥ 28	2	-0.64 [-0.83, -0.45]	0.540	0
< 28	3	-1.48 [-3.17, 0.21]	< 0.001	95.00
Duration				
≥6 months	2	-0.44 [-1.08, 0.21]	< 0.001	95.20
<6 months	4	-0.76 [-2.02, 0.51]	< 0.001	98.80
Number of participant				
>60	3	-0.81 [-1.37, -0.25]	< 0.001	85.20
≤60	3	-0.45 [-1.05, 0.15]	< 0.001	95.30

Abbreviations: PG, plasma glucose; NGR, normal glucose regulation; IGT, impaired glucose tolerance; DM, diabetes mellitus; SUA, serum uric acid; BMI, body mass index.

In our analysis, we found that (1) FPG was significantly decreased in high dosage group (allopurinol ≥ 300 mg/d) (WMD -0.53 mmol/L, 95%CI -0.74 to -0.32), while there was a minimal but statistically increase in the group of allopurinol < 300 mg/d (0.10 mmol/L; 95%CI 0.07 to 0.13). Moreover, the heterogeneities were significant decreased among studies in group of allopurinol ≥ 300 mg/d (I^2^ = 34.6%; P = 0.22) as well as in group of allopurinol < 300 mg/d (I^2^ = 0%; P = 0.69) (Table 3); (2) the reductions of FINS were more significant in older group (mean age ≥ 50 years) (WMD -1.47µ U/ml; 95%CI -2,31 to -0.63) and in the large number of participates group (WMD -0.77 µ U/ml; 95%CI -1.23 to -0.32) (Table S3); (3) the reduction of HOMA-IR was more remarkable in the patients with normal blood glucose (WMD -0.68; 95%CI -1.33 to -0.02), lower UA levels at baseline (WMD -0.77; 95%CI -1.06 to -0.47), older age (WMD -0.79; 95%CI -1.29 to -0.29), higher BMI at baseline (WMD -0.64; 95%CI -0.83 to -0.45), duration of treatment less than 6 months (WMD -0.81; 95% Cl -1.37 to -0.25) and in the trails with large number sample size (WMD -0.81; 95% Cl -1.37 to -0.25). Meanwhile, the heterogeneity (P = 0.35) of HOMA-IR was deceased among studies in group of allopurinol < 300 mg/d (Table S4); (4) no significant difference of any other outcome between other subgroups were observed; (5) the heterogeneities were decreased for studies of FPG and HOMA-IR in different dosage groups, studies for HOMA-IR in BMI ≥ 28 kg/m^2^ and with mean UA at baseline < 420 umol/L; (6) the improvement of HOMA-IR was more significant in individuals with lower UA or get higher BMI at baseline. In the sensitivity analyses, omission of any individual study from the meta-analysis did not significantly alter the pooled effects or heterogeneities.

### Publication bias

We found no evidence of substantial publication bias from Egger's or Begg's regression test (P > 0.05) for any outcome examined (Table S5).

**Table S5:** Evaluation of publication bias for studies included in the meta-analysis

	P value of the Begg's test	P value of the Fgger's test
**Primary outcomes**		
FPG (mmol/l)	1.000	0.079
FINS (µU/ml)	0.764	0.638
HOMA-IR	0.260	0.176
**Secondary outcomes**		
SBP (mm Hg)	0.462	0.056
DBP (mm Hg)	1.000	0.523
TG (mg/dl)	0.734	0.448
TC (mg/dl)	0.308	0.343

Abbreviations: FPG, fasting plasma glucose; FINS, fasting insulin; HOMA-IR, homeostasis model assessment of insulin resistance; SBP, systolic blood pressure; DBP, diastolic blood pressure; TG, triglyceride; TC, total cholesterol.

## Discussion

In metabolic syndrome, obesity and type 2 diabetes, the patients usually have metabolic inflammation, insulin resistance and high uric acid. Previous studies showed that insulin resistance is associated with high uric acid 6,33. In this meta-analysis, we showed that ULT decreased HOMA-IR, FINS and BP, but had no effect on FPG, HOMA-β or serum lipids. Of note, further stratified analyses indicated that high dosage of uric acid-lowering agents decreased FPG. These results indicate that uric acid lowering treatment may improve glucose metabolism.

In this present study, we demonstrated that hypouricemic therapy could improve insulin resistance in patients with hyperuricemia. It has been reported that hyperuricemia is an independent risk of T2DM and closely associated with IR in observational studies^9^, ^10^, ^14^, ^19^,^34^ as well as meta-analyses^10^, ^34^. Serum UA is an indirect reflection of intracellular urate, which is postulated to be the direct cause of IR.^35^ Several mechanisms might account for it. Animal experiments showed that 4 week-treatment with allopurinol in rats with hyperuricemia induced by fructose-feeding restored insulin sensitivity significantly^36^. It had been reported that high uric acid (HUA) induces IR by inhibiting insulin signaling, including inhibition of phosphorylatipn of Akt (Ser473) response to insulin and increased phosphor-insulin receptor substrate 1 (Ser307). This effect may be mediated by the generation of abnormal amounts of reactive oxygen species (ROS), as antioxidant N-acetylcysteine blocked HUA-induced activation of insulin receptor substrate 1 (IRS-1) and inhibition of Akt phosphorylation^3^, ^6^. Furthermore, a recent study also reported that elevated serum xanthine oxidase activity, but not UA cncentration, was associated with an increased risk of developing T2DM^37^, and xanthine oxidase inhibition reduced inflammatory adipokine levels and improved stress-induced insulin sensitivity^38^. Meng et al. also showed that IR was higher in primary gout patients as compared with normal individuals, and was mitigated after 12 week-hypouricemic treatment^39^. It is interesting to note that the stratified analyses showed that the reduction of IR was more remarkable in the patients with older age and in the trails with large sample size and normal blood glucose, as well as in patients received the less duration of treatment in our study. It has been reported that serum levels of uric acid were increased with age^9^, ^40^–^44^ and hyperuricemic patients usually company with relatively higher central obesity, serum lipids and BP, which were closely associated with IR^14^, ^15^, ^42^, ^45^, ^46^. Hence, IR reduction by hypouricemic treatment in patients with older age could be more significant as it was reported older patients who received anti-hyperuricemic agent were more likely to reach SUA goal^47^. Dose-response analysis showed the risk of type 2 diabetes was increased by 6% per 1 mg/dl increment in SUA level^10^. Hare et al. showed that patients with higher SUA levels benefited more after receiving uric acid lowering treatment, and post hoc analysis showed that the possible reason is that patients with high uric acid group received moe significant reductions in uric acid after treatment^48^, ^49^. IR was associated with various conditions such as obesity, inflammation, mitochondrial dysfunction, hyperinsulinemia and lipotoxicity/hyperlipidemia^50^, ^51^. It is obvious that the hyperuricemic patients with normal glucose levels had less risk factors for IR than those with IGT and diabetes, hypouricemic treatment therefore was more effective in improvement of IR in the patients with normal glucose than in those with IGT or diabetes as indicated in our study. We also showed the reduction of IR was more significant in patients received less duration of treatment. Actually, all these 4 studies with less duration of treatment were conducted in hyperuricemic patients with normal glucose, and this is why the reduction of IR was more significant in the patients.

Our study showed that FINS was significantly reduced with SUA and subgroup analyses revealed that FINS was further reduced in older individuals and in the trials with the large sample size. These results suggest that ULT improves insulin sensitivity, which is agreeable with above results. Actually, Meng et al. also found that significant higher levels of FINS and HOMA-IR in gout patients than those in the controls, but no significant difference was found regarding FPG^39^. Although FPG didn't be significantly reduced in our primary analysis, stratified analyses showed that FPG were decreased in the trials with allopurinol ≥ 300 mg/d and these trials, actually, had lower heterogeneities. These results may suggest that hyperuricemic therapy ameliorates β cell function. In vitro studies showed that hyperuricemia may contribute to abnormal glucose metabolism by causing oxidative damage and function inhibition of pancreatic β cells^5^. An animal study suggested that hyperuricemia even could cause pancreatic β-cell death and dysfunction through nuclear factor-KB (NF-kB) signaling pathway, and the deleterious effects can be attenuated by allopurinol^4^. It was also demonstrated that allopurinol reduced the additional pro-inflammatory and anti-angiogenic responses to excess glucose through its inhibition of both IL-1β and ROS production by the trophoblast^52^. Furthermore, ULT with febuxostat for 12 weeks reduced the levels of FPG in the patients with hyperuricemia^43^. These results suggest that ULT could improve β-cell function. However, our results did not show that lowering of uric acid had any effect on HOMA-β. This may be due to inadequate trials for analyses. More studies are need for future analyses.

In addition, several studies also suggested that IR itself or compensatory hyperinsulinemia may lead to hyperuricemia and this seems to be a vicious circle.^14^, ^53^, ^54^ It is, therefore, important for hyperuricemic patients to lower uric acid levels regarding its potential risk for diabetes.

The findings of our study indicated that hypouricemic treatment may has a beneficial effect on BP, which could reduce cardiovascular risk. Recent cross-sectional and cohort studies have identified hyperuricemia is closely related with the development of hypertension^55^–^59^. Moreover, a randomized, double-blind, placebo-controlled, crossover trial demonstrated that treatment with allopurinol and probenecid both resulted in significant reductions of BP, which means that uric acid was associated with increased BP that can be mitigated by ULT regardless by inhibition of uric acid production or acceleration of uric acid excretion^59^, ^60^. Actually, agreeable with our study one meta-analysis also showed that ULT decreased both SBP and DBP^61^. The possible mechanisms that high uric acid-associated hypertension may be due to high levels of uric acid trigger arteriosclerosis since oxidative stress occurred during uric acid production, urate transporter disorders, and vascular disorders from hyperuricemia^55^. Weisman et al^62^ also found that ULT was associated with reduced mortality and cardiovascular outcomes, which may attributable to the alleviation in BP.

Concerning serum lipids, our results suggested that uric acid lowering had no contribution to lipid metabolism. Several trials suggested that HUA is associated with TG and high-density lipoprotein-cholesterol (HDL-c), but not with TC or low-density lipoprotein-cholesterol (LDL-c) 34, 63, 64. However, in our study, there is no statistically decrease neither in TG nor in TC levels with the reduction of uric acid. Interestingly, Kushiyama et al discovered that oral administration of allopurinol to ApoE knockout mice markedly ameliorated lipid accumulation and calcification in vivo, but serum lipid levels were not signifcantly altered by allopurinol65. Future physiological and prospective studies are needed for the causal links and underlying mechanisms between ULT with glucose and lipid metabolism.

## Strengths and limitation

Previous studies showed that hyperuricemia is associated with insulin resistance and increases risk for diabetes^6^,^33^. Our meta-analysis is the first one to demonstrate that uric acid lowering improved insulin resistance. Secondly, the study involved with 7 eligible trials with 503 participants and 4 of them had high or medium quality, and we did detailed analysis of the limitation of each trial. No evidence of substantial publication bias in all of these trials was founded. Finally, stratified analyses provided further evidence for our conclusion as we state above.

There are several limitations for the current study. Firstly, our study did not show that hypouricemic treatment improved β cell functions although insulin resistance did be ameliorated. The main reason for this is due to limited number of trials containing the data for evaluation of β cell functions. Secondly, in our study the publications included for meta-analysis were limited by the search strategy which aims at FPG, FINS and insulin resistance, but not at serum lipid or BP, which may lead to the in-complete evaluation of data for the cardiovascular risk factors. This may partially explain several results of these outcomes in our study are different from others^27^, ^30^, ^63^. Thirdly, the heterogeneity was observed in the present study. This may be due to following reasons: (1) most of these study didn't aim to evaluate the effect of insulin sensitivity or β-cell function, thus generated some confounding factors, such as different treatment on diabetes; (2) different intervention methods in studies, such as different kinds or dosage of uric acid-lowering agents; (3) sample size was not large enough.

## Conclusion

Our meta-analysis of RCTs provided the evidence that uric acid-lowering in hyperuricemia patients significantly improved insulin sensitivity and lowered BP. Furthermore, the observation that ULT decreased fasting insulin levels in combination with improvement of insulin resistance may suggest that uric acid-lowering agents can ameliorate β-cell function. This may provide a new regimen for diabetes prevention and treatment, especially for diabetic patients with asymptomatic hyperuricemia.

## Data Availability

The datasets used and/or analysed during the current study are available from the corresponding author on reasonable request.

## Abbreviations

BP: blood pressure; CBM: Chinese Biomedical Literature Database; CI: confidence interval; DBP: diastolic blood pressures; FPG: fasting plasma glucose; FINS: fasting insulin; HDL-c: high-density lipoprotein-cholesterol; HOMA-β: homeostasis model assessment of β-cell function; HOMA-IR: homeostasis model assessment of insulin resistance; HUA: high uric acid; IR: insulin resistance; IRS-1: antioxidant N-acetylcysteine blocked HUA-induced activation of insulin receptor substrate 1; PRISMA: Preferred Reporting Items for Systematic Reviews and Meta-Analyses; RCTs: randomized controlled trials; SBP: systolic blood pressures; ROS: reactive oxygen species; SUA: serum uric acid; T2DM: type 2 diabetes mellitus; TC: total cholesterol; TG, triglyceride; ULT: urate lowering therapy; WMD: weighted mean differences.

## Findings

This study received no specific funding for this work.
